# Environmental Transmission of Typhoid Fever in an Urban Slum

**DOI:** 10.1371/journal.pntd.0004212

**Published:** 2015-12-03

**Authors:** Adam Akullian, Eric Ng’eno, Alastair I. Matheson, Leonard Cosmas, Daniel Macharia, Barry Fields, Godfrey Bigogo, Maina Mugoh, Grace John-Stewart, Judd L. Walson, Jonathan Wakefield, Joel M. Montgomery

**Affiliations:** 1 University of Washington, School of Public Health and Community Medicine, Department of Epidemiology, Seattle, Washington, United States of America; 2 Kenya Medical Research Institute, Center for Global Health Research (KEMRI-CGHR), Kenya; 3 Division of Global Health Protection, Center for Global Health, Centers for Disease Control and Prevention-Kenya, Nairobi, Kenya; 4 Departments of Global Health, Medicine, Pediatrics and Epidemiology, University of Washington, Seattle, Washington, United States of America; 5 University of Washington, Department of Statistics and Biostatistics, Seattle, Washington, United States of America; University of Queensland School of Veterinary Science, AUSTRALIA

## Abstract

**Background:**

Enteric fever due to *Salmonella* Typhi (typhoid fever) occurs in urban areas with poor sanitation. While direct fecal-oral transmission is thought to be the predominant mode of transmission, recent evidence suggests that indirect environmental transmission may also contribute to disease spread.

**Methods:**

Data from a population-based infectious disease surveillance system (28,000 individuals followed biweekly) were used to map the spatial pattern of typhoid fever in Kibera, an urban informal settlement in Nairobi Kenya, between 2010–2011. Spatial modeling was used to test whether variations in topography and accumulation of surface water explain the geographic patterns of risk.

**Results:**

Among children less than ten years of age, risk of typhoid fever was geographically heterogeneous across the study area (p = 0.016) and was positively associated with lower elevation, OR = 1.87, 95% CI (1.36–2.57), p <0.001. In contrast, the risk of typhoid fever did not vary geographically or with elevation among individuals less than 6b ten years of age.

**Conclusions:**

Our results provide evidence of indirect, environmental transmission of typhoid fever among children, a group with high exposure to fecal pathogens in the environment. Spatially targeting sanitation interventions may decrease enteric fever transmission.

## Author Summary

Typhoid fever, a serious bloodstream infection caused by the bacterium *Salmonella* Typhi, is commonly associated with direct, person-to-person transmission as a result of improper hygiene and unsafe food/water handling practices. Recent evidence, however, suggests that individuals may be *indirectly* exposed to typhoid through contact with fecal contamination in their immediate environment. In this study we investigated the role of environmental sources in the transmission of typhoid fever across an urban slum in Kenya by mapping the occurrence of cases in both children and adults. We tested the hypothesis that cases (relative to non-cases) cluster in low elevation areas as a result of the downstream flow and accumulation of fecal waste. We found that cases of typhoid fever among children tended to be concentrated in the downstream area. In adolescents and adults, on the other hand, there was little evidence of a geographic pattern in the risk of typhoid fever. These results provide evidence that environmental transmission of typhoid fever contributes to the risk of disease in children but not adults and adolescents, an observation most likely attributed to the fact that children are more likely to be exposed to fecal contamination through outside play. Interventions to improve local sanitation may therefore provide particular benefit to children who are at most risk of exposure to and acquisition of typhoid fever from environmental sources.

## Introduction

Typhoid fever is a systemic, enteric disease caused by *Salmonella enterica* serovars Typhi and Paratyphi and has an estimated annual global incidence of 26.9 million cases, and causes 200,000 deaths per year [1]. Morbidity and mortality due to typhoid fever occurs primarily in young children in Africa and Asia [2, 3]. Children lack natural immunity and experience high levels of exposure to fecal pathogens [4]. If untreated, the case fatality can exceed 10%, although appropriate antibiotic treatment can reduce case fatality to 1% or less [5].

Transmission of typhoid fever depends primarily on direct contact with the stool of an infected individual [6–8], and risk is highest in densely populated areas that lack proper sanitation and access to safe drinking water [3, 9]. Household-level hygiene and food/water safety and handling practices, as well as close contact with an index case, are associated with the direct transmission of typhoid in endemic areas [9–11]. Because *S*. Typhi is exclusively human host-adapted, reservoirs of infection exist solely within groups of infected humans, a small number of which (1–6%) develop a chronic carrier state [12, 13], which has allowed the disease to persist during inter-epidemic periods [14].

Recent evidence suggests that environmental reservoirs of infection may also support disease transmission. The risk of typhoid fever is associated with environmental factors, including proximity to open sewers and highly contaminated water bodies, residence in low elevation areas, and rainy season [3, 15–17]. Major outbreaks of *S*. Typhi have been linked to contaminated municipal water sources, and suggest waterborne transmission as an important environmental pathway [10, 18]. Whether environmental sources contribute to endemic transmission during non-outbreak periods is unclear.

There is limited study on the epidemiology and environmental drivers of *S*. Typhi infection in Africa [19], where the incidence in some urban areas parallels that of high burden regions of Asia [2]. Additional data are needed to investigate the role that environmental reservoirs play in the endemic transmission of typhoid fever in Africa, particularly among children who are at an elevated risk of infection [2]. Such information can be used to predict where risk is greatest and can inform targeting for vaccination programs, water and sanitation improvements, or other community interventons [20].

We utilized a spatial modeling framework with climatic and remotely sensed data to estimate the geographic distribution of typhoid fever risk among a large disease surveillance cohort in Kibera, a densely populated, urban informal settlement in Nairobi, Kenya. We examined the contribution of environmental exposures to transmission by testing for associations between typhoid fever risk and variations in the hydrologic landscape. These data suggest that environmental transmission is an important contributor to the risk of typhoid fever in young children but may not be important in adults and adolescents.

## Methods

### Ethical statement

The protocol was reviewed and approved by the Institutional Review Boards of the United States Centers for Disease Control and Prevention (US-CDC) and the Kenya Medical Research Institute (KEMRI). All data analyzed were anonymized to ensure confidentiality of the study participants.

### Study Area

Kibera is an informal urban settlement with between 250,000–500,000 residents in Nairobi, Kenya [21]. The area lacks adequate sanitation infrastructure, as indicated by open sewers and limited access to clean water [22], and has a large burden of many infectious diseases, including an adult HIV prevalence at 12.6% [23]. The wet season in Kibera is characterized by long rains from March–May and short rains from October–November; dry season runs from June–September and December–February. The average monthly temperature is 19°C [24].

**Fig 1:**
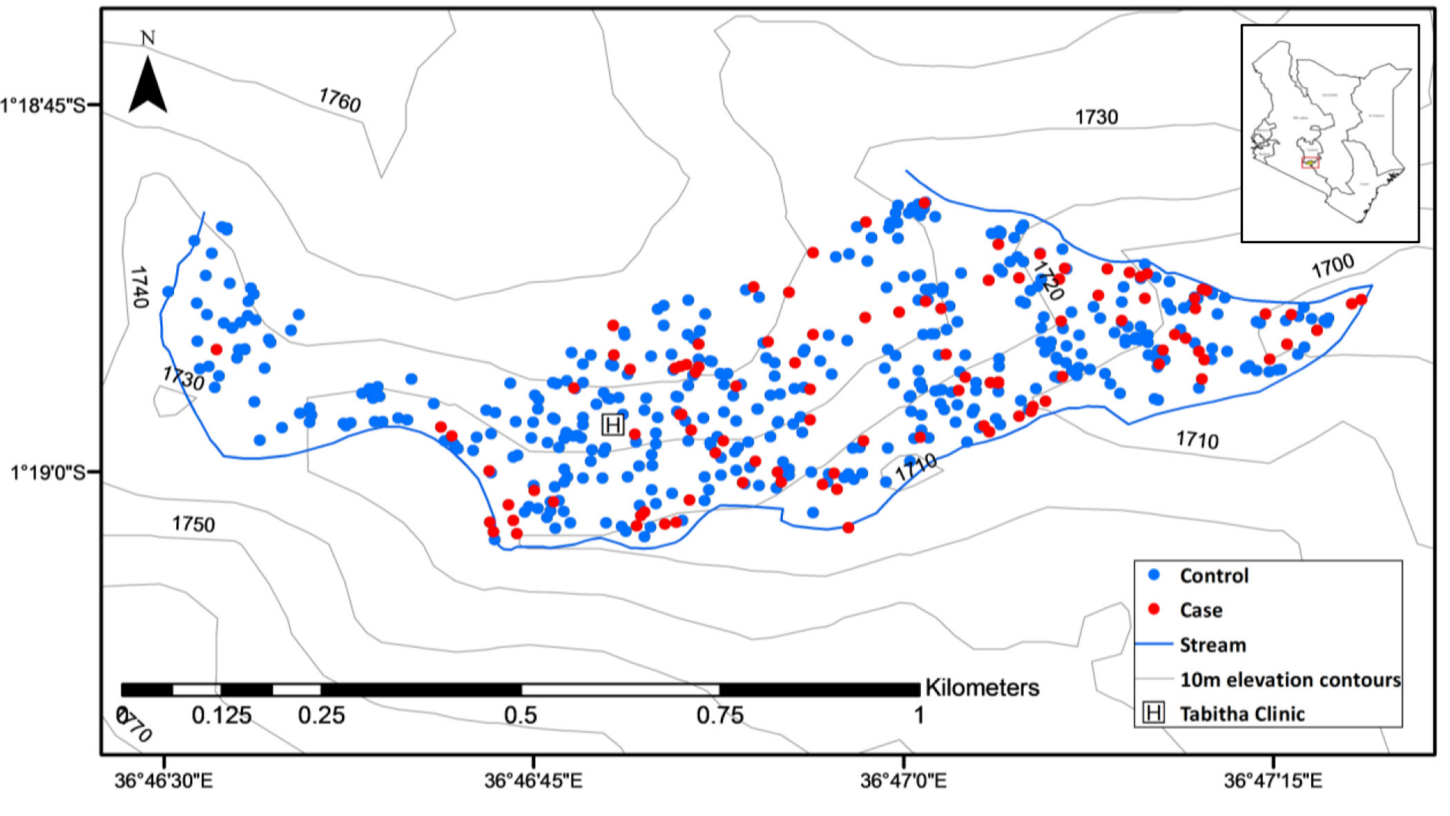
Spatial distribution of 110 cases and 440 controls between 2010–2011 in Kibera, Kenya.

Disease surveillance data were obtained from an ongoing, KEMRI/CDC-Kenya, population-based, household and clinic surveillance system in Kibera. Details of the surveillance have been described previously [2, 24]. Briefly, about 28,000 individuals have been followed biweekly since 2006 by trained community interviewers, and those with fever during these household visits were advised to seek medical attention at the surveillance site clinic. Blood cultures were conducted on all consenting individuals presenting to the clinic with an axillary temperature of ≥38.0 degrees C. The surveillance area covers 0.40 km^2^, has a high population density (70,000 individuals/km^2^) [2], and lies at an altitude of 1700–1740 meters, with terrain gently sloping towards the Motoine River in the southeastern portion of the study area (Fig 1).

### Case Selection

Incident cases of symptomatic typhoid fever were ascertained between Jan 1, 2010 and Dec 31, 2011 at Tabitha Clinic, the primary surveillance clinic in the study area providing free care to those enrolled in the Kibera cohort. Tabitha Clinic is centrally located within 600 meters of the entire study population. Cases were defined as individuals who presented to Tabitha Clinic with acute febrile illness ≥ 38°C and from whom *S*. Typhi was isolated through blood culture. Only the first episode of typhoid fever during the 2-year study period was included for any individual with more than one occurrence. Cases whose residential location could not be verified 14 days prior to a typhoid fever episode were excluded from the analysis.

### Control Selection

Controls were randomly selected from the underlying surveillance cohort (~28,000 individuals) in order to estimate the geographic distribution of the population at risk. A pool of control households with verified GPS coordinates was compiled for the years 2010–2011 (study years) and all individuals residing in those households during that time were eligible for control selection if they met the following criteria: 1) not identified as a case of typhoid fever between 2010 and 2011 (whether or not fever had been reported), 2) enrolled in the study with at least one household study visit between 2010 and 2011, and 3) non-missing GPS coordinates at the time of study visit. Four controls per case were selected at random, and one study visit from each control was randomly selected from the series of study visits. The rationale for selecting controls randomly was to estimate the spatial distribution of the underlying population at risk. Controls were not matched to cases on potential confounders such as age and location in order to allow for analysis of those co-variates.

### GIS and Hydrological Variables

Handheld Garmin GPSMap76CSx units were used to capture Global Positioning Systems (GPS) points on case households 14 days prior to diagnosis (to account for the average incubation period), and at the time of interview for the control households. Household elevation was estimated for each case and each control using a 90 X 90 meter digital elevation model (DEM) downloaded from NOAA.gov [25]. The DEM was used to generate a continuous 90 X 90 meter flow accumulation surface using the ArcHydro Toolset in Arc GIS 10.0 [26], with values at each location corresponding to the upstream contributing area draining to that point (e.g., lower-values are higher in the watershed and have less upstream area draining to that location, whereas high-value cells are lower in the watershed and have more upstream contributing area). DEM data are freely available, provide good approximations of the dominant flow direction of surface water at the watershed scale [27], and have been used to model the hydrologic diffusion and geographic distribution of different infectious diseases [28–31]. We used DEM-derived elevation and flow accumulation surfaces to approximate the level of exposure to accumulated fecal contamination at each location across the study area. We also measured the Euclidean distance from each case/control to the nearest point along two heavily polluted streams that bound the study area to test for associations between typhoid fever risk and exposure to point source contamination along the streams.

Rainfall data recorded at a weather station at Nairobi’s Jomo Kenyatta International Airport (JKA) between 2010 and 2011 were downloaded from https://data.noaa.gov/dataset/global-surface-summary-of-the-day-gsod, “The Global Surface Summary of Day” product, produced by the National Climatic Data Center (NCDC) [32]. The total accumulation of rainfall over 3, 7, and 30 day periods was calculated 14 days prior to each case diagnosis (to account for the average incubation period for typhoid following exposure) and 14 days prior to each control interview for comparability.

### Statistical Analysis

We conducted a spatial case-control analysis to identify geographic and environmental risk factors for *S*. Typhi infection. We compared the distribution of demographics (age, gender, household size) and environmental/hydrological variables (elevation, flow accumulation, distance to stream, and daily rainfall) between cases and controls in both univariate and multivariate logistic regression models. Multivariate analyses were stratified on age group (children over ten and adults/adolescents under ten years of age) in order to test for differential effects of environmental risk factors on typhoid transmission in each age-stratified risk group. Children under ten years of age experience the highest incidence of typhoid fever in our study area [2], and may also have unique exposure pathways that warrant further investigation. Multivariate analyses were adjusted for confounding factors identified *a priori*. All logistic regression analyses were performed using STATA 11 (STATACo, Texas 77845 USA).

### Spatial Analysis

We used spatial regression models with logit links to compare the geographic distribution of cases relative to controls among groups of individuals over and under 10 years of age, adjusting for the spatial confounding effects of individual-level co-variates. Under the null hypothesis, the distribution of cases relative to controls is expected to be equivalent to the age-group-specific case-control ratio at every location across the study area. Maps of the adjusted odds were produced for each age group (> and < 10 years) using a locally weighted regression smoother in a general additive model (GAM) framework for case-control data [33] using a logistic link function and a non-parametric component for the residual spatial surface:

$$Logit(P)=\;\alpha_{0}\;+\;\beta_{1}(\;x_{1})+\;\beta_{2}(\;x_{2})+\;S(lat,lon)$$

Where *P* is the probability of being a case versus control, *x*_1_ is age in years, *x*_2_ is the number of individuals in the household, and *S* is a thin plate spline used to smooth latitude and longitude over the geographic extent of the study area. We adjusted for individual-level factors in the model (age and household size) to estimate the residual spatial surface and test for its significance. When plotted on a map, the residual spatial surface is expressed as the residual log-odds of being a case versus control–where a value of zero corresponds to the expected case-control ratio. A log-odds above (below) zero indicates a larger (smaller) than expected proportion of cases to controls. We tested for significance of the log-odds surfaces against the null hypothesis of no spatial heterogeneity. Multiple transmission events within the same household were considered independent observations and were thus modelled without adjusting for spatial autocorrelation at the household level. The residual spatial surface models both small scale household-level transmission events as well as larger scale environmental heterogeneity in risk.

All spatial analyses were performed in the R-statistical software [34] using the mgcv package for fitting GAMs (methods described elsewhere [35]) and visualized using the splancs package[36]. GAM plots were stratified on age greater than and less than ten years.

## Results

### Study population

A total of 118 cases of typhoid fever were confirmed at Tabitha clinic between Jan 1, 2010 and Dec 31, 2011. Of those, 111 had confirmed residence and household-level GPS coordinates at the time of infection and were included in the analysis. One case was excluded as it was a repeat episode of typhoid on the same individual within a one month period, which we consider a recrudescent case as opposed to two separate transmission events. The 110 incident cases included were unique individuals who resided in 103 unique households, with 7 secondary cases within the same household. Among the 7 secondary cases at the household level, 5 occurred within 30 days of an index case in the same household, which we classify as intra-household transmission events. Four hundred and forty controls were randomly selected from the underlying population at risk, comprising 416 households, with interview dates spanning from January 6, 2010 to December 29, 2011.

### Comparison of socio-demographic characteristics of cases and controls

**Fig 2:**
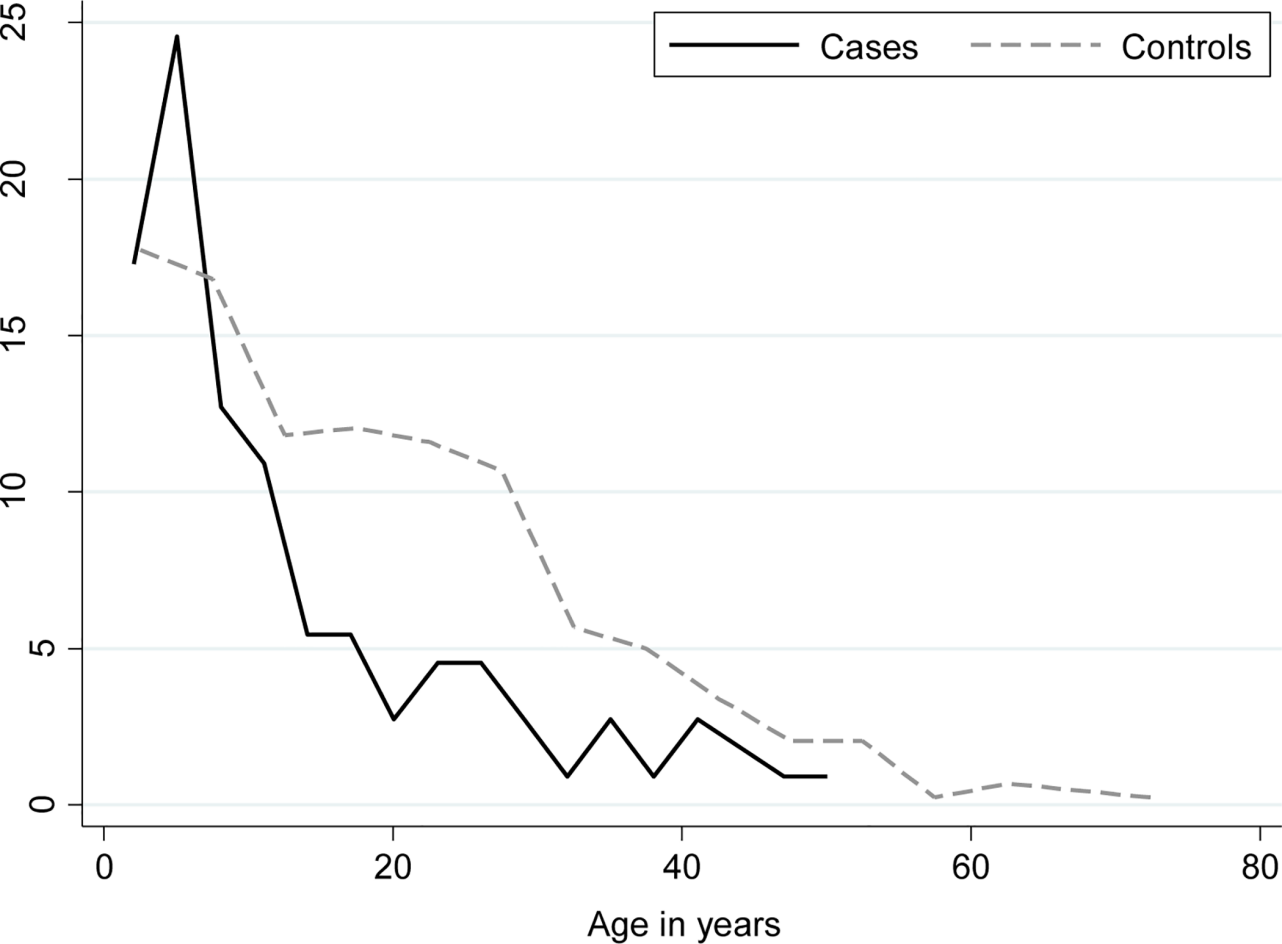
Distribution (by percentage of total) of 110 typhoid fever infections and 440 population-based controls by age in Kibera.

The distribution of demographic and environmental characteristics between cases and controls is shown in Table 1. Cases were significantly younger than controls (mean age of 8.4 years versus 16.4 years, respectively, (p<0.001). More than half of cases (56.4%) were under age ten versus 34.6% of controls (p<0.001, Fig 2). Cases resided in households with more individuals (70.0% of cases lived in households with more than 5 individuals compared to 48.6% of controls, p<0.001). There was no evidence that cases differed from controls with respect to gender (52.7% female versus 49.8% female, respectively, p = 0.579).

**Table 1:** Demographic and environmental variables by case-control status.

	Cases		Controls		
	n = 110		n = 440		
	Number (percent)		Number (percent)		p-value^1^
**Age (median/IQR)**					
0–4 years	29	(26.4)	78	(17.7)	**<0.001**
5–9 years	33	(30.0)	74	(16.8)	
10–14 years	16	(14.6)	52	(11.8)	
15–24	15	(13.6)	104	(23.6)	
25+	17	(15.5)	132	(30.0)	
**Gender**					
(% female)	58	(52.7)	219	(49.8)	0.580
**Number of inhabitants in household**					
1–2	0	(0.0)	23	(5.2)	**<0.001**
3–5	33	(30.0)	203	(46.1)	
>5	77	(70.0)	214	(48.6)	
**Season** ^2^					
Wet	45	(40.9)	213	(48.4)	0.160
Dry	65	(59.1)	227	(51.6)	
**Distance to Tabitha Clinic (meters)**					
< 100	6	(5.5)	56	(12.7)	**0.019**
100–500	64	(58.2)	257	(58.4)	
> 500	40	(36.4)	127	(28.9)	
**Elevation (meters)** ^3^					
1,695–1,707	21	(19.1)	55	(12.5)	**0.007**
1,708–1,720	41	(37.3)	111	(25.2)	
1,721–1,733	21	(19.1)	143	(32.5)	
1734–1,747	27	(24.6)	131	(29.8)	
**Flow accumulation** ^4^					
0	30	(27.3)	164	(37.3)	**0.001**
1–5	38	(34.6)	176	(40.0)	
6–10	5	(4.6)	21	(4.8)	
> 10	37	(33.6)	79	(18.0)	
**Distance to stream (meters)**					
0–24	34	(30.9)	74	(16.8)	0.078
25–99	45	(40.9)	242	(55.0)	
> 100	31	(28.2)	124	(28.2)	
**Total precipitation in past three days (inches)** ^5^					
No precipitation	75	(68.2)	287	(65.2)	0.205
< 0.1	14	(12.7)	41	(9.3)	
0.1–0.4	11	(10.0)	49	(11.1)	
> 0.4	10	(9.1)	63	(14.3)	

^1^ Based on linear test of trend for ordered categorical variables

^2^ Determined by approximate month when exposed (e.g., diagnosis date minus 14 days to account for typhoid fever incubation period). Wet season is characterized by long rains from March–May and short rains from October–November. Dry season runs June–September and December–February.

^3^ Measured using Shuttle Radar Topography Mission Digital Elevation Model (DEM) data with 90 meter resolution

^4^ A 90 meter resolution hydrological surface generated from a Digital Elevation Model (DEM), used to estimate the amount of accumulated flow draining to every location in the study area.

^5^ Measured as the sum of all rainfall over a three day window occurring 14 days (the incubation period for typhoid) prior to either date of diagnosis for cases or interview date for controls

Comparison of spatial, temporal, and climatic characteristics of cases and controls

**Fig 3:**
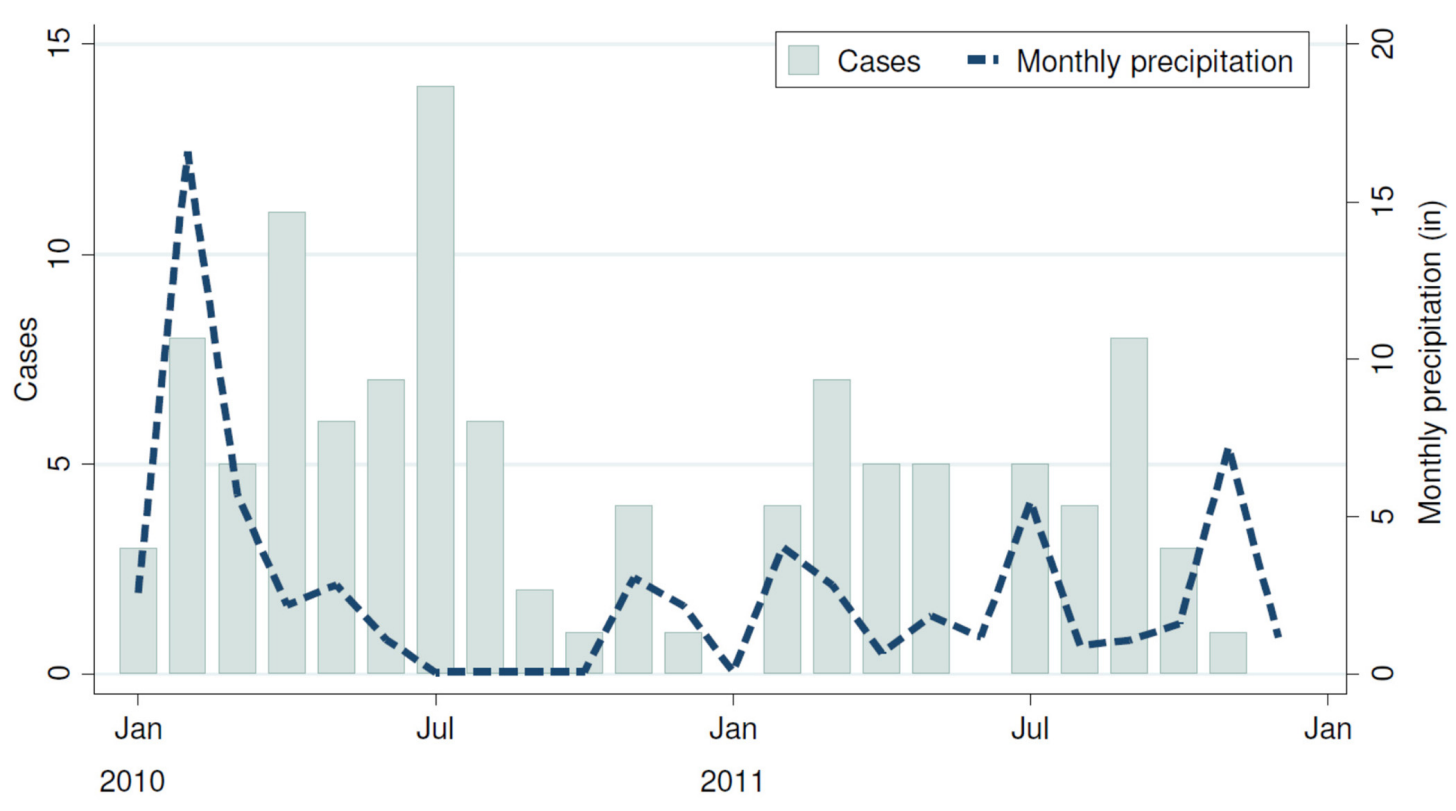
Monthly distribution of 110 incident typhoid cases between 2010–2011 in Kibera with monthly precipitation overlaid.

The spatial distribution of the 110 incident cases and 440 controls is displayed in Fig 1. Compared to controls, cases were concentrated in the eastern, lower elevation region of the study area. Only one case was observed in the westernmost part of the study area. The incidence of typhoid fever over the 2-year study period did not follow any seasonal pattern, nor was there any discernable association between monthly rainfall and risk (Fig 3).

Individuals with typhoid fever resided in lower elevation areas (19.1% of cases resided in the lowest elevation area, 1,695–1,707 meters, compared to 12.5% of controls, p<0.001) with higher flow accumulation (33.6% of cases resided in areas with the highest flow accumulation area compared to 18.0% of controls, p<0.001). There was no evidence that cases differed from controls with respect to season (43.6% of cases occurred in the wet season versus 51.1% of controls, p = 0.161), or total precipitation in the three days prior to infection/interview (68.2% of cases and 65.2% of controls occurred after three days with no precipitation, p = 0.205).

### Age-stratified analyses

**Fig 4:**
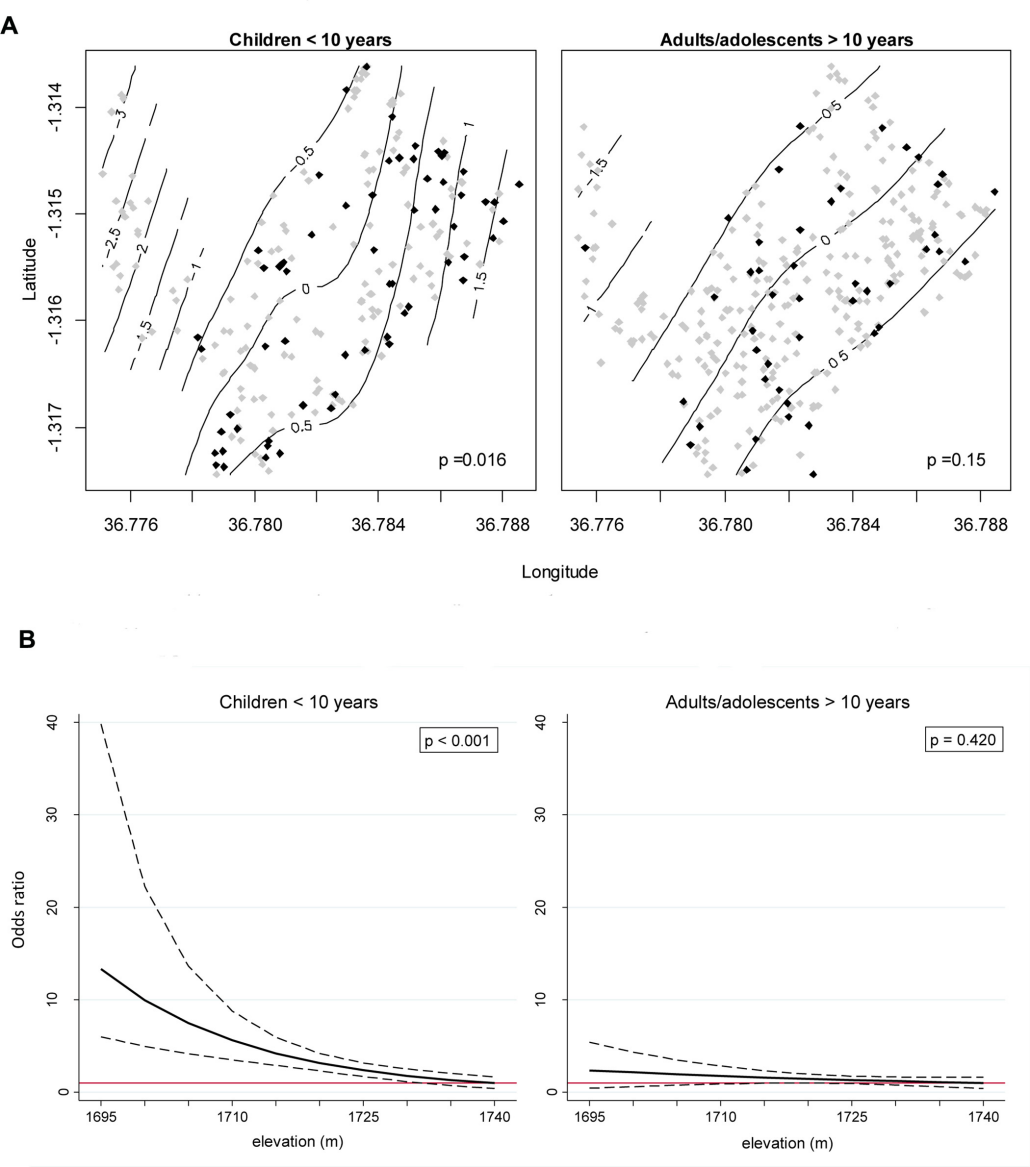
(A) Smoothed log odds (contours) of typhoid fever by age group across the study area, adjusted for age and household size, with cases (black points) and controls (grey points) overlaid. P-values test for significance of the observed spatial pattern in log odds against a homogenous risk surface (i.e., where the log odds is zero at all locations). (B) Adjusted odds ratios from the multi-variate logistic model (Table 2) for the association between elevation and typhoid fever for both children (<10 years) and adults/adolescents (>10 years), with 95% CI overlaid (dashed lines). Odds ratio of 1 (ref odds = 1740 meters in elevation) indicated by horizontal red line.

Household size (measured as number of inhabitants) was positively associated with risk of typhoid in both children (under age ten) and adults/adolescents (over age ten), OR = 1.27, 95% CI (1.11–1.46), p < 0.001; OR = 1.20, 95% CI (1.10–1.31), p < 0.001, respectively. Among children under age ten, those who resided at lower elevation had significantly greater risk of typhoid fever compared to those in higher elevation areas, OR = 1.87, 95% CI (1.36–2.57), p <0.001, corresponding to a ten meter decrease in elevation (Table 2), shown graphically in Fig 4B. Similarly, those children who resided in areas with higher flow accumulation had greater risk of typhoid fever compared to children in areas with less flow accumulation, OR = 1.27, 95% CI (1.00–1.62), p = 0.05. Among adults/adolescents over age ten, there was no evidence of an association between typhoid risk and elevation, OR = 1.23, 95% CI (0.89–1.71), p = 0.205 or flow accumulation, OR = 1.11, 95% CI (0.91–1.37), p = 0.305. Long periods of dryness (>30 days of no rain) prior to exposure/interview were positively associated with higher risk of typhoid in adults/adolescents, OR = 2.88, 95% CI (1.05–7.87), p = 0.040], with no evidence of an association in children.

**Table 2:** Adjusted odds ratios by demographic and environmental variables.

	Children < 10 years				Adults/adolescents > 10 years			
	Cases *N (%)*	Controls *N (%)*	aOR (95% CI)	p-value	Cases *N (%)*	Controls *N (%)*	aOR (95% CI)	p-value
**Demographics**								
Age (years)^1^	62	152	1.02 (0.91–1.15)	0.695	48	288	0.98 (0.95–1.01)	0.125
Household size (# inhab.)^1^	62	152	**1.27 (1.11–1.46)**	**<0.001**	48	288	**1.20 (1.10–1.31)**	**<0.001**
**Environment**								
Distance to streams (m)^1^	62	152	1.00 (1.00–1.01)	0.199	48	288	1.00 (0.99–1.01)	0.471
Elevation (10m decrease)^1^ ^,^ ^2^	62	152	**1.87 (1.36–2.57)**	**<0.001**	48	288	1.23 (0.89–1.71)	0.205
Flow accumulation^3^	62	152	**1.27 (1.00–1.62)**	**0.050**	48	288	1.11 (0.91–1.37)	0.305
**Climate** ^4^								
Season (dry) ^4^	34 (54.8)	75 (49.3)	1.24 (0.68–2.26)	0.467	31 (64.6)	152 (52.8)	1.63 (0.86–3.08)	0.131
No precip. in prev. 3 days^4^	36 (58.1)	100 (65.8)	0.65 (0.34–1.24)	0.196	39 (81.3)	187 (64.9)	2.13 (0.96–4.74)	0.063
No precip. in prev. 30 days^4^	5 (8.1)	7 (4.6)	1.67 (0.49–5.73)	0.411	7 (14.6)	14 (4.9)	**2.88 (1.05–7.87)**	**0.040**

^**1**^Adjusted for age, household size, elevation, and distance to streams (excluding co-variate of interest)

^**2**^DEM Shuttle Radar Topography Mission Digital Elevation Model, (expressed as per 10 meter decrease in elevation)

^3^Upstream contributing area in units of 10 90X90 square meters. Adjusted for age, household size, and distance to streams

^4^Calculated from 14 days prior to case diagnosis or control interview to account for the incubation period. No adjustment

Among children less than ten years of age, the risk of typhoid fever was geographically heterogeneous across the study area (p = 0.016) and generally followed a linear geographic gradient, with risk increasing from the west to east (Fig 4A). There was a weaker, and non-statistically significant (p = 0.150) spatial pattern in the risk of typhoid fever among adolescents/adults greater than ten years of age.

## Discussion

In this large, population-based, case-control study, risk of typhoid fever was spatially heterogeneous across a small geographic area. The observed spatial pattern in risk was especially pronounced among children under ten years of age, who experienced an almost doubling of risk for every ten meter decrease in elevation. In contrast, the spatial distribution in the risk of typhoid fever among adults and adolescents varied only slightly across the study area. Our results suggest distinct modes of transmission of typhoid fever between children and adolescents/adults in a Kenyan urban slum. Transmission in children may be more environmentally mediated than that in adults.

Low elevation areas aggregate bacterial pathogens from diffuse sources upstream via the overland flow of surface waters [37–42], and may serve as environmental reservoirs for a number of water-borne and water-related infectious diseases in children, including soil transmitted helminthes and schistsomiasis [30]. Our results build on evidence from previous studies that have shown environmental heterogeneity in the risk of typhoid fever [16, 43] by highlighting differences in environmental drivers of risk between children and adults.

The global health implications of environmental transmission of typhoid fever among children in Africa are significant. Young children are at the greatest risk of typhoid fever in densely populated urban areas with poor hygiene and sanitation infrastructure [2, 9]. Unlike adults and adolescents, children have underdeveloped natural immunity conferred by previous infection and are unable to fight systemic bacterial colonization [44]. Furthermore, young children are likely to be exposed to fecal contamination in the immediate environment surrounding their household [45]. Based on estimates from 2006–2009 in Kibera, children between 2–4 years of age and 5–9 years of age experienced the highest incidence of typhoid fever (2242.6 cases per 100,000 person years and 1,788 per 100,000 person years, respectively), compared to 821.5 cases per 100,000 person years among 0–1 year olds. The higher incidence among children two and over is consistent with behaviors associated with outdoor play and exposure to fecal pathogens in the environment. Based on our results, it is plausible that in typhoid-endemic areas children contract *S*. Typhi from environmental reservoirs at substantially higher rates than do adults. Environmental transmission may therefore account for a large part of the burden of typhoid fever in children and may in turn play an important role in its continued transmission.

We observed a negative association between precipitation and risk of typhoid fever in adults/adolescents greater than ten years of age, who experienced a 3-fold higher risk of infection after long dry periods. The effect of rainfall on risk of enteric pathogens, including typhoid fever, is unclear. Heavy rainfall is associated with increased risk of enteric disease transmission as a result of washing fecal pathogens into drinking water sources [46, 47]. One study from Nepal showed an increase in typhoid fever incidence in parallel with seasonal peaks associated with the rainy season [15]. On the other hand, long dry periods may increase the risk of diarrheal pathogens as a result of the limited availability of clean water for proper hygiene [48, 49].

A primary strength of our study was the use of population-based controls to estimate the underlying geographic distribution of the population at risk. The selection of controls in a clinic or hospital-based setting, a practice used in many spatial epidemiology studies, can obscure true disease-exposure associations if the controls do not adequately represent the geographic distribution of the underlying population at risk. A second strength is the stratification of our data into under and over ten years of age, which allowed us to compare the effects of environment on typhoid transmission across age groups.

The results of our study must also be considered in light of certain limitations. First, we lacked individual-level socioeconomic status (SES), which is needed to adjust for small-scale variation in access to resources, improved sanitation, and hygiene practices. Considering that our population was restricted to a small geographic area with a relatively homogenous distribution of SES and sanitation, we do not expect strong confounding by SES across the study area. Second, though Tabitha Clinic was the primary point of care for individuals with syndromes consistent with Typhoid fever (60–70% of individuals with acute fever seek care at Tabitha Clinic), there are inevitably missed cases [2]. Not all individuals with symptoms consistent with Typhoid in the cohort uptake care at Tabitha Clinic. We do not, however, expect bias due to residential proximity to the clinic. Tabitha’s location is closer to the upstream, low incidence area than the downstream, high incidence area and any confounding by proximity to Tabitha would therefore attenuate our estimates towards the null. Next, though DEM-derived surfaces have been used to model hydrological drivers of waterborne and water-related infectious diseases [30, 50], they are limited in their ability to capture fine scale heterogeneity in the flow of water and thus only crudely measure the accumulation of water-borne contamination. Finally, our study did not confirm environmental transmission via bacteriologic evidence indicating the presence of *S*. Typhi in the environment.

In summary, this study provides evidence of environmental transmission of typhoid fever in young children in an urban slum in Africa. Implementation of interventions to reduce transmission should include targeted sanitation improvements in areas of high geographic risk, particularly in low elevation areas where fecal waste tends to concentrate. Children living in these areas are at increased risk of typhoid transmission and may benefit from environmental interventions and targeted vaccination campaigns, as has been emphasized previously [2, 20].
