# Correction: Environmental Transmission of Typhoid Fever in an Urban Slum

**DOI:** 10.1371/journal.pntd.0004353

**Published:** 2016-01-04

**Authors:** The PLOS Neglected Tropical Diseases Staff

In the Results section of the Abstract, the last sentence contains a typo. The sentence should read as the following: In contrast, the risk of typhoid fever did not vary geographically or with elevation among individuals more than ten years of age.
